# Single-cell transcriptomic analysis of the tumor ecosystem of adenoid cystic carcinoma

**DOI:** 10.3389/fonc.2022.1063477

**Published:** 2022-11-17

**Authors:** Quanquan Lin, Zhanjie Fang, Jinlong Sun, Fei Chen, Yipeng Ren, Zhenhong Fu, Sefei Yang, Lin Feng, Feng Wang, Zhigang Song, Wei Chen, Wenjun Yu, Chen Wang, Yixin Shi, Yue Liang, Haizhong Zhang, Hongzhu Qu, Xiangdong Fang, Qing Xi

**Affiliations:** ^1^ Department of Stomatology, Chinese People's Liberation Army General Hospital, Beijing, China; ^2^ Key Laboratory of Genome Sciences and Information, Beijing Institute of Genomics, Chinese Academy of Sciences/China National Center for Bioinformation, Beijing, China

**Keywords:** adenoid cystic carcinoma, head and neck cancer, single-cell transcriptomic analysis, MYB, EN1

## Abstract

Adenoid cystic carcinoma (ACC) is a malignant tumor that originates from exocrine gland epithelial cells. We profiled the transcriptomes of 49,948 cells from paracarcinoma and carcinoma tissues of three patients using single-cell RNA sequencing. Three main types of the epithelial cells were identified into myoepithelial-like cells, intercalated duct-like cells, and duct-like cells by marker genes. And part of intercalated duct-like cells with special copy number variations which altered with MYB family gene and EN1 transcriptomes were identified as premalignant cells. Developmental pseudo-time analysis showed that the premalignant cells eventually transformed into malignant cells. Furthermore, MYB and MYBL1 were found to belong to two different gene modules and were expressed in a mutually exclusive manner. The two gene modules drove ACC progression into different directions. Our findings provide novel evidence to explain the high recurrence rate of ACC and its characteristic biological behavior.

## 1 Introduction

Adenoid cystic carcinoma is a kind of malignant tumor of the exocrine glands. The annual incidence of salivary gland ACC has been reported to be 0.16 to 0.14 per 100,000 populations (1). Compared with other solid tumors, ACC is characterized by more aggressive behavior, perineural invasion, early pulmonary metastasis, and a higher rate of positive incision edge (2).The current clinical treatment for ACC is surgery and adjuvant radiotherapy but recurrence or metastasis still occurs in more than 50% ACCs (3). The survival rates of 5 years, 10 years, and 20 years is 68%, 52%, and 28% respectively, indicating the bad prognosis (4).

Histopathologically, ACC is a type of epithelial tumor comprised of ductal and myoepithelial cells. The expression of MYB is the gold standard for diagnosis of ACC (4, 5). With the widespread use of whole exome sequencing (WES) and whole genome sequencing (WGS), its internal oncogenic mechanism has been gradually revealed, such as the MYB family gene translocation (6), the Notch signal pathway (7), and the DNA damage repair (8) and epigenetic molecular mutation pathways (9). However, the underlying molecular mechanisms of tumor development remain unexplained clearly.

Single-cell RNA sequencing (scRNA-seq) (10) can be used to observe the evolution of individual cells in various paracarcinoma and carcinoma tissues. It is widely used in breast cancer (11), ovarian cancer (12), non-small-lung cancer (13), pancreatic ductal adenocarcinoma (14), and other cancers. Nevertheless, scRNA-seq is poorly reported in ACC.

In this study, we analyzed the epithelial cell clusters in the paracarcinoma and carcinoma tissues of three patients and aimed to elucidate the mechanisms of ACC at the single-cell transcriptional level. Our findings revealed the possible origin of ACC cells and mapped the progression of tumor development at the transcriptional level.

## 2 Results

### 2.1 Overview of cell population in the ACC tumor ecosystem

**Figure 1:**
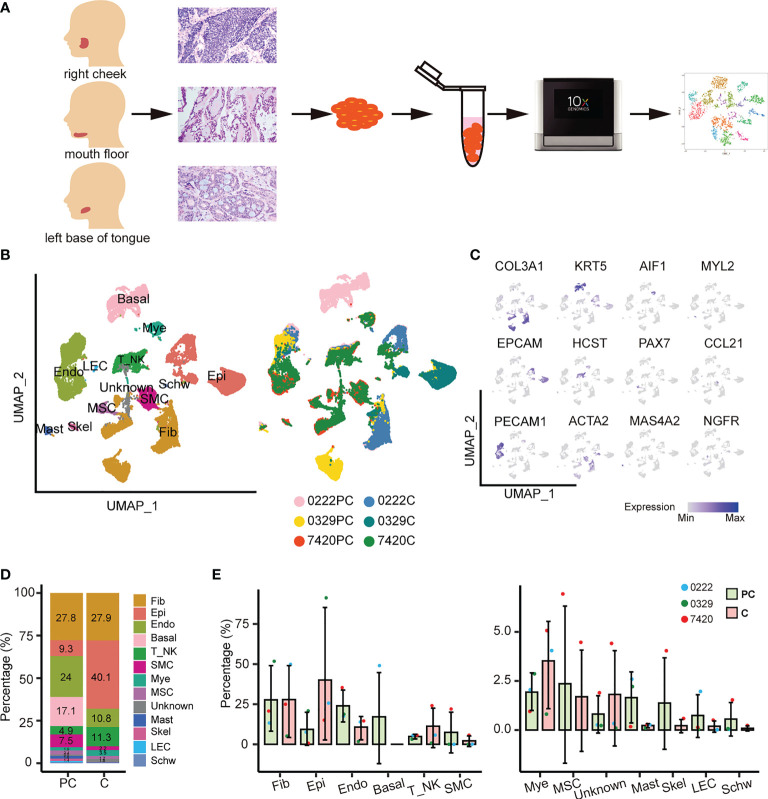
Overview of THE ACC tumor microenvironment. **(A)** Workflow for collecting clinical samples and processing scRNA-Seq data. **(B)** UMAP of 13 cell populations from six samples. PC, paracarcinoma; C, carcinoma. **(C)** Cell subtypes were labeled in the UMAP plot using typical markers. **(D)** The abundance of each cell population in the paracarcinoma and carcinoma tissues. **(E)** The relative abundance of 13 cell clusters in the paracarcinoma and carcinoma tissues. Error bars are presented as mean values ± SD.

The tumor ecosystem of ACC was examined using scRNA-seq of digested living cells derived from ACC paracarcinoma and carcinoma tissues using a 10x Genomics-based platform (**Figure 1A**). A total of 49,948 cells from the parcarcinoma and carcinoma tissues of three ACC patients were captured using the sequencer and 42,714 cells were retained for downstream analysis after quality control filtering (**Supplementary Table 1**). The results of the staining pathologic sections of three patients are shown in **Supplementary Figure 1**. The clinicopathological characteristics of three patients are listed in **Supplementary Table 2**.

Unsupervised clustering of the cells identified 13 cell types, including fibroblasts, epithelial cells, endothelial cells, basal cells, T or natural killer cells, smooth muscle cells, myeloid cells, muscle satellite cells, mast cells, skeletal muscle cells, lymphatic endothelial cells, Schwann cells, and an unknown cell cluster, which was a mixture of plasma cells, B cells, and fibroblasts (**Figure 1B** and **Supplementary Figure 2B**).

Uniform manifold approximation and projection (UMAP) visualization of the hallmark genes expressed in each cell subtype was performed (**Figure 1C**). The dot plots show the well-expressed marker genes in corresponding cell types (**Supplementary Figures 2A, C**). The complex tumor ecosystem comprised 40.1% epithelial cells, 27.9% fibroblasts, 11.3% T/NK cells, 10.8% endothelial cells, and a small number of other cells (**Figure 1D**). Notably, basal cells only existed in sample 0222 paracarcinoma tissue, whereas smooth muscle cells, muscle satellite cells, unknown cells, skeletal muscle cells, and Schwann cells only existed in sample 7420 paracarcinoma and carcinoma tissues (**Figure 1E**).

The proportion of cell types in the paracarcinoma and carcinoma tissues were examined. The proportion of epithelial cells was 9.3% in paracarcinoma, while in carcinoma was 40.1%. In contrast, the proportion of endothelial cells in paracarcinoma and carcinoma were 24.0% and 10.8%, respectively (**Figures 1D, E**). Considering ACC is a malignant tumor that originates from epithelial cells, the transcriptome characteristics of epithelial cells were further evaluated in the study.

### 2.2 Various subtypes of epithelial cells play different roles in tumor progression

**Figure 2:**
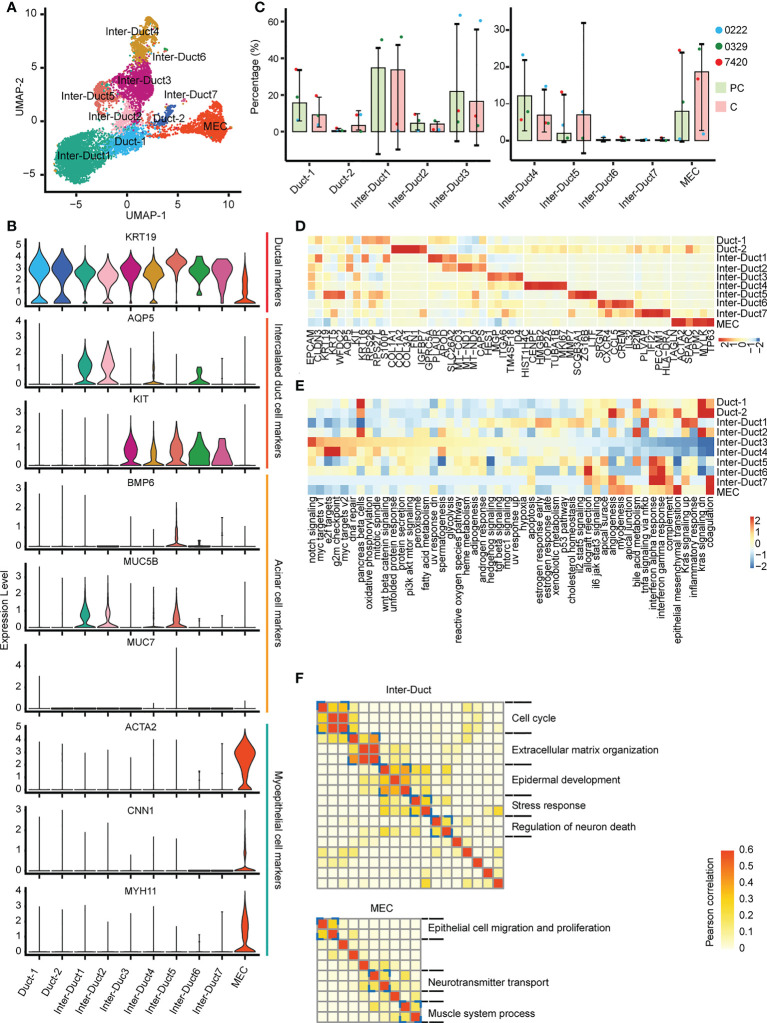
Different epithelial cell subtypes with specific functions. **(A)** UMAP visualization of 10 epithelial cell subtypes across the three patients. **(B)** Violin plots showing the expression of duct, acinar, and myoepithelial cells markers in epithelial cells subtypes. **(C)** The relative abundance of 10 epithelial cell subtypes in the paracarcinoma (PC) and carcinoma (C) tissues. **(D)** Heatmap of differentially expressed genes in epithelial cell clusters. **(E)** Heatmap showing differences in 50 hallmark pathways enrichment scores among each epithelial cell subtype. **(F)** Heatmap showing pairwise correlations of intratumoral programs derived from Inter-Duct (top) and myoepithelial-like cells (bottom). Coherent expression programs across tumors are marked on the right.

The 9,685 epithelial cells present were categorized as three subtypes (**Figure 2A**): myoepithelial-like cells (MECs, ACTA2^+^/MYH11^+^/CNN1^+^), intercalated duct-like cells (Inter-Duct 1–7, KRT19+/AQP5+/KIT+), and duct-like cells (Duct 1 and Duct 2, KRT19+/AQP5-/KIT-). MUC5B, which is a mucinous acinar marker, was expressed at lower levels in a few subtypes, such as Inter-Duct 5. Moreover, MUC7, which is a serious acinar marker, was barely expressed in any subtypes (**Figure 2B**). In the paracarcinoma tissues, Inter-Duct 1, Inter-Duct 3, Duct 1, Inter-Duct 4, and MECs accounted for 34.7%, 21.9%, 15.6%, 12.2%, and 8.0% of the epithelial cells, respectively. In the carcinoma tissues, MECs, Inter-Duct 5, and Duct 2 accounted for 18.7%, 7.0%, and 3.4% of the epithelial cells, respectively (**Figure 2C**).

Each cluster was analyzed to identify differentially expressed genes. HES1 and ID4 were highly expressed in Inter–Duct 3 cells. CENPF, TOP2A, and MK167 were highly expressed in Inter–Duct 4 cells. ACTA2 and TP63 were highly expressed in MECs. SCGB3A1, MMP7, and ZG16B were highly expressed in Inter–Duct 5 cells. SRGN, CCL5, and CREM were highly expressed in Inter–Duct6 cells. PLVAP and PECAM1 were highly expressed in Inter–Duct 7 cells (**Figure 2D**).

Gene set variation analysis (GSVA) revealed the function of the different epithelial clusters. Notch signaling pathway, MYC targets, DNA replication process, oxidative phosphorylation, Wnt β-catenin, and Pi3k-Akt-mTOR signaling pathway were upregulated in Inter-Duct 3–4 cells. Duct 2 and MECs were involved in epithelial–mesenchymal transformation, angiogenesis, myogenesis, and apical junction. Inter-Duct 5–7 cells showed high expression of immune-related genes and were enriched for pathways including interferon α/γ response, allograft rejection, and IL6 JAK STAT3 signaling (**Figure 2E**).

These results suggested that each epithelial cluster was enriched for different signaling pathways. Inter-Duct 3–4 cells were enriched for activation of tumor progression pathways and Inter-Duct 5–7 cells may be involved in the immune response.

Differences in gene expression programs of three main epithelial cell clusters were performed by non-negative matrix decomposition. Hierarchical clustering identified five expression programs that varied within the Inter-Duct cells, including cell cycle, extracellular matrix organization, epidermal development, stress response, and regulation of neuron death. Three gene expression programs were found in MECs, including epithelial cell migration and proliferation, neurotransmitter transport, and muscle system process (**Figure 2F**). We identified the dominant gene sets in the different expression programs of three main epithelial cell clusters and performed GO (Gene Ontology) enrichment analysis on specific genesets to support above findings (**Supplementary Table 3**, **Supplementary Figures 3, 4**).

These results suggested that Inter-Duct cells may be directly involved in tumor development and myoepithelial cells play a contributory role in this process.

### 2.3 The underlying regulatory network in epithelial cell clusters

The potential molecular basis driving the distinct epithelial clusters was examined using SCENIC (Single-cell Regulatory Network Inference and Clustering) to identify the underlying regulatory network in epithelial cells.

**Figure 3:**
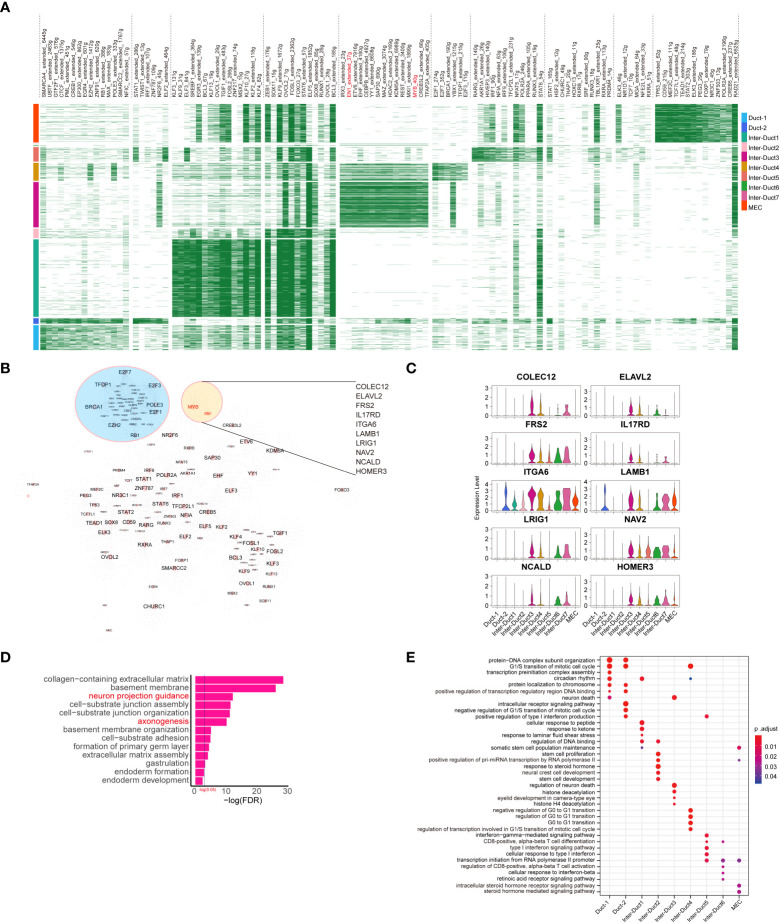
Development of epithelial cell subtypes is driven by distinct TFs. **(A)** Heatmap showing differentially expressed transcriptional regulons in different epithelial cell subtypes. **(B)** Epithelial cell transcription factor regulatory network, of which significant clusters or regulons with their targets are labeled. **(C)** Violin plots showing the relative expression of downstream target genes coregulated by MYB and EN1 in each epithelial cell subtype. **(D)** The result of GO enrichment analysis for target genes coregulated by MYB and EN1 and genes interacting with them. Biological processes terms associated with the peripheral nervous system are highlighted in red font. **(E)** Dot plot showing the results of GO enrichment analysis for predominant TFs of different epithelial cell subtypes.

Each cluster was driven by different transcription factors (TFs). Notably, coexpression of MYB and EN1 was observed in the upstream TFs of Inter-Duct 3–4 cells (**Figure 3A**). In addition, genes involved in DNA repair and cell cycle transition, such as E2F2, TFDP1, E2F3, BRCA1, POLE3, E2F1, EZH2, and RB1, showed aggregation (**Figure 3B**). Prediction of genes downstream of MYB and EN1 identified 10 coregulated downstream target genes, including COLEC12, ELAVL2, FRS2, IL17RD, ITGA6, LAMB1, LRIG1, NAV2, NCALD, and HOMER3 (**Figure 3C**), which are predominantly associated with the nervous system (15–21). Genemania (22) was used to predict possible functions coregulated by MYB and EN1, which are involved in cell–substrate junctions and neuronal guidance. (**Figure 3D**)

Gene Ontology (GO) enrichment analysis of the TFs drove different expression programs. Duct 1 and Duct 2 cells were enriched for G1/S transition of the mitotic cell cycle, protein–DNA complex subunit organization, and intracellular receptor signaling pathway. Inter-Duct 3 cells were enriched for regulation of neuron death, histone deacetylation, and eyelid development in camera-type. Inter-Duct 4 were enriched for negative regulation of G0/G1 transition, cell cycle regulation, and transcription regulation involved in the G1/S transition of the mitotic cell cycle. Inter-Duct 5 cells were enriched for cellular response to type I interferon and transcription initiation from RNA polymerase II promoter. MECs were enriched for maintenance of the somatic cell population and steroid hormone-mediated signaling pathway (**Figure 3E**). These results indicate that Inter-Duct 3 cells were regulated by the upstream TFs, MYB and EN1.

### 2.4 Copy number variation in epithelial cells derived from paracarcinoma and carcinoma tissues

**Figure 4:**
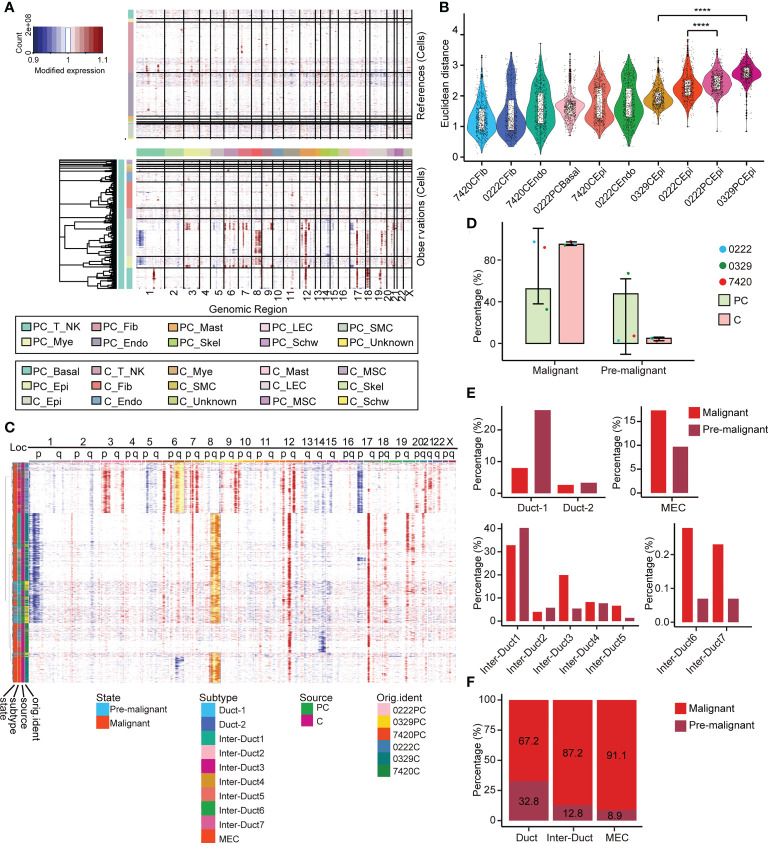
Multilayer heterogeneity of CNVs in epithelial cells. **(A)** Nonmalignant cells were used as references (top) and large-scale CNVs were observed in epithelial and basal cells (bottom). **(B)** A total of 600 epithelial cells, endothelial cells, basal cells, and fibroblasts were randomly selected from each corresponding sample and the Euclidean distance between the CNV score of which and the median CNV score of fibroblasts in adjacent samples was calculated and ranked respectively and presented as a scatterplot. All p-values were calculated by Wilcoxon rank sum test, **** p ≤ 0.0001. **(C)** Reclustering the CNV score matrix of the epithelial cells. Cell grouping information is annotated below. **(D)** The relative abundance of pre-M and malignant cells in PT and tumor tissues. Error bars are presented as mean ± SD. **(E)** The relative abundance of 10 epithelial cell subtypes in the pre-M and malignant cells. **(F)** The relative abundance of the three main epithelial cell populations in the pre-M and malignant cells. pre-M: pre-malignant, PC: paracarcinoma, C: carcinoma.

The large-scale chromosome CNV status of all the cells from the paracarcinoma and carcinoma tissues was examined using infercnv (**Figure 4A**). Comparing with the endothelial cells and fibroblasts from the paracarcinoma, all epithelial cells showed complex CNV changes, suggesting a malignant tendency in most cells (**Supplementary Figure 5A**). Moreover, different clusters of epithelial cells from the paracarcinoma tissue underwent the massive copy number amplification in 6q, 8q, 12q, and 17p, and chromosomal deletions in 14q (**Figure 4C**). We assume that the majority of tumor tissue derived from epithelial cells with similar CNV pattern are malignant, while epithelial cells that do not conform to that pattern, which were mainly identified in the paracarcinoma tissue are considered to be premalignant (Pre-M) cells. (**Figure 4B**).

Examination of the paracarcinoma and carcinoma tissues revealed that 47.6% of epithelial cells in paracarcinoma tissues were pre-M cells (**Figure 4D**). Moreover, among the pre-M cells, 26.2% were Duct 1 cells, 40.3% were Inter-Duct 1 cells, 5.4% were Inter-Duct 3 cells, 1.4% were Inter-Duct 5 cells and 9.7% were MECs. In comparison, the proportion of Inter-Duct 3 cells in malignant cells was 19.9%, which was significantly higher than the pre-M cells 5.4%. In addition, nearly 17.3% malignant cells were MECs (**Figure 4E**). The percentage of the pre-M cells in the Duct cells, Inter-Duct cells, and MECs were 32.8%, 12.8%, and 8.9%, respectively. On the contrary, the proportion of malignant cells in each cell clusters were 67.2%, 87.2%, and 91.1%, respectively. (**Figure 4F**).

MYB and EN1 were identified as the upstream TFs and MYB was identified as the hallmark ACC gene. MYB/MYBL1/EN1 were only highly expressed in epithelial cells (**Supplementary Figure 5B**). MYB and EN1 were highly expressed in malignant Inter-Duct 3–7 cells, and MYBL1 was highly expressed in Inter-Duct 1 cells, Inter-Duct 2 cells and MECs. (**Supplementary Figure 5C**). Moreover, among three main epithelial cell types, MYB was highly expressed in malignant Inter-Duct cells, MYBL1 was highly expressed in malignant MECs, and EN1 was highly expressed in all of them (**Supplementary Figure 5E**). These findings were consistent with previous studies that reported that the expression of MYB and MYBL1 were mutually exclusive (11) (**Supplementary Figures 5C, D**).

Importantly, the stem cell gene score of pre-M cells was higher than that of malignant cells, reflecting the accuracy of our method (**Supplementary Figure 5F**).

### 2.5 Different gene modules defined the developmental trajectory states

**Figure 5:**
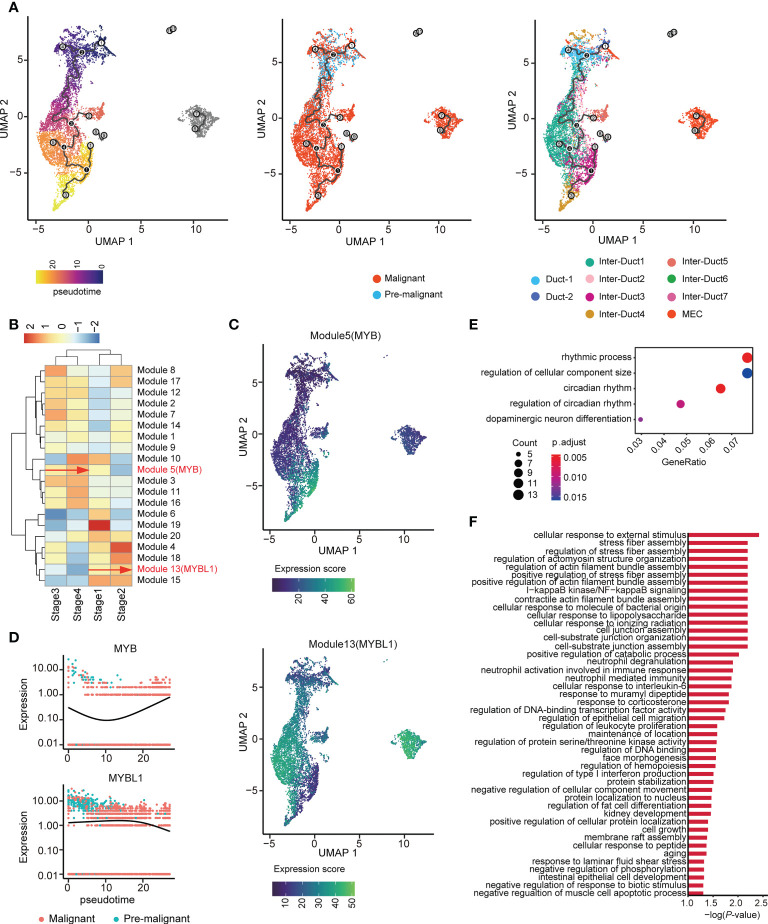
Two distinct evolutionary directions of ACC. **(A)** UMAP visualization of epithelial cells colored by pseudo-time (left), malignant state (middle), and subtype (right). **(B)** Unsupervised clustering heatmap showing the heterogeneity of gene modules expression over the pseudo-time. MYB and MYBL1 belong to Modules 5 and 13, respectively. **(C)** UMAP plot shows the gene expression score of Modules 5 (upper) and 13 (lower) among epithelial cells. **(D)** Dynamics of MYB (upper) and MYBL1 (lower) along pseudo-time. **(E)** Dot plot showing the results of GO enrichment analysis for genes in Module 5. **(F)** Bar chart showing the results of GO enrichment analysis for genes in Module 13.

Differentiation trajectory analysis was used to examine the evolution of epithelial cells. The nodes of pre-M cells with a higher stemness score were set as the root node of pseudo-time. Most of the pre-M cells were gathered initially and evolved into malignant cells by the end of the pseudo-time. Notably, MECs, which were used as an independent differentiation group, were not involved in these processes (**Figure 5A**).

The pseudo-time axis was divided into four stages according to its lower quartile, median, and upper quartile. Graph-autocorrelation analysis revealed coregulated genes in the modules within the four stages. MYB and MYBL1 were highly expressed in modules 5 and 13 (**Figure 5B**, **Supplementary Table 4**), originated from the same root node, and showed increased expression along the pseudo-time trajectory, evolving toward two completely different branches (**Figure 5C**). Consistent with the above-mentioned results, during tumor evolution, gene MYB and MYBL1 showed opposite trends in expression (**Figure 5D**).

In addition, GO enrichment analysis of these two gene modules showed that the genes in Module 5 were involved in the rhythmic process, regulation of cellular component size, and dopaminergic neuron differentiation (**Figure 5E**). Furthermore, the genes in Module 13 were associated with diverse and complex biological processes, such as cellular response to external stimulus (**Figure 5F**). Taken together, these findings indicate that genes coregulated with MYB or MYBL1 may drive the two different evolutionary directions of ACC.

### 2.6 Intercellular communications in the ACC tumor microenvironment

**Figure 6:**
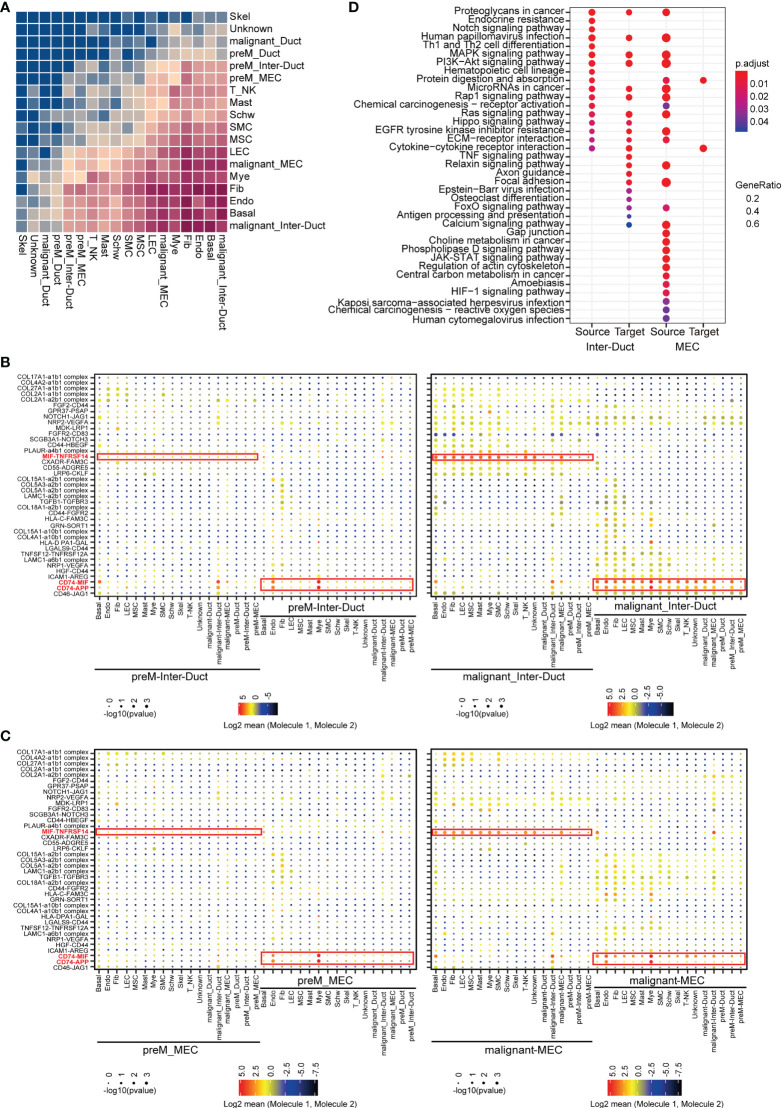
Intercellular interactions in ACC. **(A)** Heatmap showing the relative strength of cellular communication between different cell types. The depth of rectangular color is positively correlated with the number of interacting receptors-ligand pairs between cell types. **(B)** Dot plot showing the mean expression level and p-values for the selected interacting partners between other cells and intercalated duct-like cells. The interacting communication with Pre-M Inter-Duct was shown in left and those with malignant Inter-Duct was shown in right figure. **(C)** Dot plot showing the mean expression level and p-values for the selected interacting partners between other cells and myoepithelial-like cells. The interacting communication with Pre-M MEC was shown in left and those with malignant MEC was shown in right figure. **(D)** Dot plot showing the results of KEGG enrichment analysis for upregulated receptor and ligand genes in different groups, which were cell pairs with malignant Inter-Duct-like cells or MEC-like cells as “Source” or “Target”.

The tumor microenvironment is essential for the proliferation, migration, survival, anti-immune killing of malignant cells. Thus, we applied CellPhoneDB to infer cell-cell communication from combined expression of multi-subunit ligand-receptor complexes from scRNA-seq data. We found that malignant_inter_duct, Basal cells, endothelial cells, fibroblasts, myeloid cells, malignant_MEC were the dominant communication hubs (**Figure 6A**).

We classified cell communication pairs into 12 categories according to malignancy grade, predominant epithelial subset, and molecular origin. A significant upregulation in the expression of a number of receptors and ligands has been observed in malignant Inter-Duct and malignant MECs. Moreover, compared with preM cells, MIF-TNFRSF14, CD74-MIF and CD74-APP were generally highly up-regulated in the communications between malignant Inter-Duct cells or MECs and other cells.

MIF (Macrophage Migration Inhibitory Factor) plays an import dual role of pro-inflammatory and pro-oncogenic (23). TNFRSF14 is a member of tumor necrosis factor (TNF) receptor superfamily, functioning in activating inflammatory and inhibitory T-cell immune response (24). The up-regulation of MIF-CD74 were found in many cancers, such as cervical squamous cell carcinoma (25), hepatocellular carcinoma (26) and prostatic cancer (27). Studies have shown that MIF-CD74 may enhance the proliferation and inhibit the apoptosis of tumors by promoting angiogenesis of tumor microenvironment. In addition, it has been reported that MIF may be a novel prognostic marker for human oral squamous cell carcinoma (28). This suggested that inflammation and immunosuppression in tumor microenvironment may provide favorable conditions for ACC proliferation and invasion (**Figures 6B, C**).

The upregulated receptor–ligand genes in different groups were implicated in different Kyoto Encyclopedia of Genes and Genomes (KEGG) pathways strongly associated with cancerization (**Figure 6D**). Overall, the enhanced communications between malignant and stromal cells in the tumor microenvironment further indicate the complexity of tumor behavior. The tumor microenvironment is essential for the proliferation, migration, survival, and anti-immune killing of malignant cells and the combined expression of multisubunit ligand–receptor complexes identified in the scRNA-seq data may infer cell–cell communication.

## 3 Discussion

The epithelial structures of normal salivary gland tissue are divided into four parts: acinus, intercalated duct, striated duct, and excretory duct (29). However, it is difficult to distinguish between the intercalated duct cells and other duct cells at the cellular level. Intercalated duct cells have been reported to act as stem cells with the potential to differentiate into ductal cells, which has not been shown at the cellular level (30). This is the first study to identify the epithelial cells from the perspective of scRNA-seq in ACC, which is essential to further clarify its pathogenesis route.

### 3.1 Transcriptome characteristics of epithelial cells in ACC

The dynamic changes in the homologous subpopulations of the Inter-Duct 1 and 7 cells from paracarcinoma and carcinoma tissues may be related to the different biological functions of each cell subpopulation in tumor progression.

GSVA showed that each Inter-Duct cell clusters were enriched for different signaling pathway, including Notch signaling pathway, MYC, and immune response-related pathways (31, 32). The results indicated that different homologous cell clusters had different biological functions.

We used SCENIC to identify the TFs for each epithelial cell cluster. GO enrichment analysis of the dominant TFs showed that they controlled different expression programs.

MYB and EN1, as upstream TFs, regulated downstream genes related to domain neural activity together (33), histone deacetylation, and other tumor processes (34, 35) in Inter-Duct 3–4 cells. This is the first time we identified EN1 as a TF in ACC, may regulate the activity of different cell types in scRNA-seq at the transcription level.

The clustering of TFs, POLE3 (DNA Polymerase Epsilon 3), TFDP1, RB1, and E2F transcription factor families (E2F3, E2F3, and EF7) were associated with cell cycle transition (36), suggesting possible disruption of the normal epithelial cell division cycle (**Figure 3B**). Duct 1 and Duct 2 cells shared many biological processes, which were mostly related to transcription. Inter-Duct 1 cells were enriched for stress response-related pathways, such as response to peptide and laminar shear stress. Inter-Duct 2 cells were associated with stem cell proliferation and differentiation. Inter-Duct 5 and Inter-Duct 6 cells were associated with immune processes, such as T cell activation and interferon response. MECs were enriched for maintenance of somatic cell populations and steroid hormone-mediated signaling pathways (**Figure 3E**).

Our findings contribute to further understanding of the heterogeneity of biological functions by different TFs. Furthermore, these can explain the neural invasion from a scRNA-seq perspective in ACC.

### 3.2 Identified and verified premalignant cells in paracarcinoma tissues

Large-scale CNV analysis validated an intermediate state between precancer and cancer in the transcriptional profile of samples from exocrine glandular malignancies, such as breast cancer, prostate cancer, and their paracarcinoma tissues (37–39).

In our study, the complex CNVs were found in the epithelial cells from the paracarcinoma tissues. We speculated that there was an abundant population of specialized cells with abnormal transcription. They will transform into malignant cells in the paracarcinoma tissues in the future but cannot be accurately determined at the cellular or protein levels. This may be relevant to the frequently positive incisal edge in ACC. We tried to identify and define this unique cell population as pre-M cells.

Sample 0222 and 0329 showed a long arm amplification at chromosomes 6 (40) and 8 (41), respectively. As previously reported (42), MYB and MYBL1 were located at 6q23 and 8q13.1, respectively. This suggested that the expression of these two genes may affect copy number changes at the chromosomal level. In addition, translocation fusion of MYB family genes with NFIB is the gold standard for diagnosis of ACC, in which the expression rate is about 65%–85% (43). Previous study reported that EN1 was a potential biomarker for worse prognosis in ACC (44). This may explain the association of EN1 with poor prognosis from the single cell transcriptome level.

The distribution of the pre-M and malignant cells in each cell cluster was observed.

We attempted to calculate the stemness gene score of pre-M and malignant cells as it may be useful to determine the potential for transforming cells from pre-malignant to malignant states. Assuming that about 95% of the epithelial cells in carcinoma tissues are malignant, we observed a relatively equal proportion of pre-M and malignant cells in paracarcinoma tissues. This may suggest that pre-M cells may transform into malignant cells. Comparing with the different cluster, more malignant cells were found in Inter-Duct 3, which activated tumor-associated signaling pathways by upstream TFs, MYB and EN1.

Subsequently, MYB family genes and EN1 were expressed in both pre-M and malignant Inter-Duct cells. MYB was previously shown to be highly expressed in only duct cells by immunohistochemistry and fluorescence *in situ* hybridization (45). Notably, the stemness gene score of pre-M was higher, suggesting its potential cancer transforming characteristics. These findings fully validate the pre-M cells in the paracarcinoma tissues. It can be a more accurate explanation for the false-negative incision edge and high recurrence rate from a single-cell transcriptome perspective in ACC.

### 3.3 Pseudo-time analysis of epithelial cell trajectory development from pre-M to malignant cells

The abnormal manifestation of complex CNVs verified the presence of specific pre-M cells in the paracarcinoma tissues and the expression of MYB verified its accuracy. We used scRNA-seq pseudo-time analysis to infer the differentiation trajectory of the epithelial cells and evolution of the cell subtypes to explain how the pre-M cells eventually become malignant throughout tumor progression.

The pseudo-time was divided into four stages. At the beginning of the time, a considerable number of cells were pre-M, which was mainly enriched in the Duct 1 cluster. As the timeline progressed, they gradually transformed into malignant cells and overlapped with Inter-Duct 3/4/6. Inter-Duct 3–4 cells were regulated by the TFs, MYB and EN1, and underwent activation of tumor-associated pathways. Myoepithelial cells were separated from the rest of the epithelial cell population and similarly transformed over time into a malignant cell population. This finding is fully consistent with the pathological features of ACC.

Graph-autocorrelation analysis revealed that MYB gene expression was progressively enhanced during the transition from stage 3 to 4, whereas MYBL1 showed high gene expression during the transition from stage1 to 2. UMAP (Uniform Manifold Approximation and Projection) analysis of the pseudo-time showed that MYB and MYBL1 showed exclusive expression at the end of the proposed time, which is consistent with the findings of previous studies (41, 46). GO enrichment analysis of these two gene modules showed that these two modules may drive two different evolutionary directions of ACC. This could explain the further development of ACC under the regulation of MYB homologous genes from a single-cell transcriptional perspective.

To sum up, the study is the first to report the use of scRNA-seq to examine ACC at the transcriptome level. We identified a special population of Inter-Duct cells. Inter-Duct 3–4 cells were coregulated by the upstream TFs, MYB, and EN1, which were enriched for tumor progression pathways. In addition, pre-M cells were found in paracarcinoma tissues, which was verified by the presence of MYB gene, and were highly expressed mainly in malignant Inter-Duct cells, including Inter-Duct 3–7. In addition, MYBL1 was highly expressed in malignant Inter-Duct and myoepithelial cells. Finally, pseudo-time analysis revealed that different cell clusters eventually transformed from a pre-M to malignant state in the ACC progression. Two modules containing MYB and MYBL1 drove different trajectories. Our findings further explain the high recurrence rate and unique biological characteristics of ACC.

## 4 Materials and methods

### 4.1 Ethical statement

The present study was approved by the Ethics Committee of Chinese People's Liberation Army (PLA) General Hospital and was performed according to the guidelines of the Declaration of Helsinki. Written informed consent was obtained from each patient prior to sample collection.

### 4.2 Human specimens

Preoperatively, the CT data of patients were imported into the Robotic-assisted navigation system. The surgeon could locate the tumor precisely by the robot. We removed the paracarcinoma and carcinoma tissues by enlargement. Samples were obtained from the paracarcinoma and carcinoma tissues of three ACC patients who underwent surgery at the Chinese PLA General Hospital Stomatology department. The primary foci were on the minor salivary gland. The sample 7420 and 0329 were both cribriform types, in addition sample 0222 was cribriform type in 2019 and recurrence to be solid type in 2021 in pathology.

The tissues from sample 0222 for scRNA-seq was incised for recurrence in 2021. And then we compared pathological sections of 0222 samples between 2019 and 2021 for further study. Each sample was carefully reviewed by two experienced pathologists to confirm the pathology. Single-cell data information of the six samples in shown in **Supplementary Table 1**. The clinical data are summarized in **Supplementary Table 2**.

### 4.3 Cell preparation for scRNA-seq

#### 4.3.1 Tissue collection

The patients who underwent the surgery received the informed consent for the specimen. Preoperative head skin preparation was performed on the three patients and robotic positioning patches were applied on surgery morning. The CT data were scanned and imported into the robotic navigation system. After the patients were successfully intubated through the nasal cavity, the Mayfield 3-peg head frame was placed, and after checking the stability of each mechanical joint fixation. The robot was matched with the patient’s head positioning marker points and the navigation system was activated after successful fusion. Under the precise guidance and positioning of the navigation robot, the patient’s paracarcinoma and carcinoma tissues were removed. A part of the tissue obtained during surgery was placed in 10% neutral formalin solution at room temperature and sent to pathology for definitive patient diagnosis. The other tissues were submerged in 4°C tissue preservation solution and put into ice box for immediate transport to the laboratory for single cell transcriptome study.

#### 4.3.2 Tissue dissociation

Tissue samples were cut into small pieces of around 1 mm^3^ in size and placed in petri dish and covered with PBS (Gibco). Each sample was then transferred to centrifuge tube with adding 2mL digestive system (Adult Brain Dissociation Kit, mouse and rat NO.130-107-677), 750μL Enzyme mix 1 (Enzyme P 50μL and Buffer Z 1900μL) and 30μL Enzyme mix 2 (Buffer Z 20 μL and Enzyme A 10μL). After running the gentle MACS Program m _brain_01 program on the tissue. the tissues were incubated in water bath at 37°C for 15 min, and then filtered the cell suspension, centrifuged at 300×g for 10 min at 4°C to completely remove the supernatant.

The samples were left to stand for 2–3 min and collected the supernatant. The supernatant was removed, and the cells were resuspended in red blood cell lysis buffer, according to the ratio of cell suspension to red blood cell lysate at 1:3, and incubated for 2–3 min at room temperature prior to centrifugation at 120× *g* at 4 °C for 3 min. Finally, the cells were resuspended in PBS.

### 4.4 10X Genomics scRNA-Seq

#### 4.4.1 Cell capture and cDNA synthesis

Cell capture and cDNA synthesis were performed using a Chromium Single-Cell 3’ Gene Expression library and Gel Bead Kit V3.1 (10x Genomics, 1000075). Cell profiling was performed using a Single-Cell B Chip Kit (10x Genomics, 1000074). Cell suspension containing 300–600 living cells/μL (determined using Count Star) was loaded onto the Chromium single-cell controller (10x Genomics) to generate single-cell gel beads in the emulsion according to the manufacturer’s protocol. In brief, single cells were suspended in PBS containing 0.04% bovine serum albumin.

Around 8,700 cells were added to each channel with a targeted cell recovery estimate of 8,000 cells. Captured cells were lysed and the released RNA was barcoded through reverse transcription in individual GEMs (Gel bead-In-EMulsions). GEMs were reverse transcribed in a C1000 Touch Thermal Cycler (Bio Rad) programmed at 53°C for 45 min, 85°C for 5 min, and held at 4°C. After reverse transcription, single-cell droplets were broken, and single-stranded cDNA was isolated and cleaned with Cleanup Mix containing DynaBeads (Thermo Fisher Scientific). cDNA was generated and amplified, and the quality was assessed using the Agilent 4200.

#### 4.4.2 Preparation of the scRNA-Seq library

Single-cell RNA-seq libraries we reconstructed using Single-Cell 3’ Library and Gel Bead Kit V3.1 according to the manufacturers’ instructions. The libraries were finally sequenced using an IlluminaNovaseq6000 sequencer with a sequencing depth of at least 100,000 reads per cell with a pair-end 150 bp (PE150) reading strategy.

#### 4.4.3 Processing of scRNA-Seq data

Raw data produced from the 10× Genomic platform were processed by Cell Ranger (v6.1.2) (47) and mapped to the human reference genome GRCH38. Pre-processed data were imported into R (v4.1.2) and analyzed using Seurat (v4.0.3) (48). The quality control thresholds for the number of genes detected and the proportion of mitochondrial and hemoglobin transcripts were the mean plus 2.58-fold standard deviation across all cells. As a supplement, in a single cell, the number of genes detected needed to be >200, and the ratio of mitochondrial genes and hemoglobin genes had to be <30 and 10, respectively. Cells that did not meet these criteria were discarded. A linear equation according to the corresponding table of the number of loading cells and the multiplet rate provided by 10× company was fitted. Then the doublets were identified with appropriate multiplet rates from the linear equation above by DoubletFinder (v2.0.3) (49) and will be removed subsequently. Each sample was subjected to quality control separately to ensure that high-quality cells were remained. The top 2,000 variable features were chosen for PCA, and the 50 most significant PCs were selected for subsequent cluster analysis. All cell types were manually identified and further examined by R package singleR (v1.6.1) (50). Epithelial cells were extracted and then batch effect across different samples was removed using the Harmony algorithm in R package harmony (v0.1.0) (51) before identifying neighbors and finding clusters.

### 4.5 Marker genes for cell populations

Differentially expressed genes were identified using the FindAllMarkers function of the Seurat package for each subcluster. Specific genes were selected to serve as a basis for artificially defining cell populations as follows (52–58): fibroblasts (COL3A1, DCN, COL1A1, LUM, COLA2, COL6A2, FBN1), epithelial cells (EPCAM, KRT19, CLDN3 and KRT8), endothelial cells (PECAM1, ENG, VWF), basal cells (KRT5, KRT14, TP63), T/NK cells (GZMA, HCST), smooth muscle cells (ACTA2, MYH11, ACTG2, CNN1, CALD1, MCAM, TAGLN, PDGFRB, MYL9), myeloid cells (AIF1, CD163, LYZ), muscle satellite cells (PAX7, CD82, NCAM1, MYF5), mast cells (MS4A2, TPSB2, GATA2), skeletal muscle cells (ACTA1, NEB, MYL2), lymphatic endothelial cells (PDPN, PROX1, LYVE1), Schwann cells (NGFR, SOX10, GAP43, CDH19), plasma cells (JCHAIN, IGKC, IGHA1, IGHA2), T cells (CD3G, CD3D, CD3E), and B cells (MS4A1, BANK1, CD37).

### 4.6 Identification of malignant cells

Large-scale chromosomal copy number alterations within cells were detected using the R package inferCNV (v1.8.1). Immune and stromal cells from paracarcinoma tissues were used as presumptive “normal” cells as a reference, and their CNV scores were set as baseline. A previously described method was used to distinguish malignant cells from all epithelial cells (59). In brief, 1,600 fibroblasts and endothelial cells were stochastically picked from paracarcinoma samples, among which 1,000 were regarded as a reference and the remaining cells and epithelial cells were considered as an observation. CNV analysis was performed using inferCNV. When most of the spiked “normal” stromal cells were gathered in a specific cluster on a dendrogram, the other cells in this cluster were considered as “normal epithelial cells”. Correspondingly, cells not belonging to this cluster were identified as “malignant epithelial cells.” Since only 29 epithelial cells were identified as normal epithelial cells, accounting for only 0.003% of all epithelial cells, we concluded that all epithelial cells show malignant characteristics. Epithelial cells with malignant features were further subclassified. The median CNV estimates of all fibroblasts in the paracancerous samples were defined as the baseline, and the Euclidean distance between the CNV estimates of all observation cells and the above baseline was calculated. We determined the range of Euclidean distances (median ± 2 SD) by assuming that most epithelial cells (95%) in the carcinoma samples were “malignant” in the true sense, whereas cells whose distance from the baseline was not within this range were considered pre-M cells. The signature genes used to distinguish subtypes of epithelial cells or to calculate the score of stemness of different class of epithelial cells are as follows (60–62): intercalated duct cells (KIT or AQP5), myoepithelial cell genes (ACTA2, MYH11, CNN1), and stemness genes (ALDH1A1, CD44, PROM1, NANOG, KIT, NES, KLF4, CD55, ALCAM, NOTCH4, WNT7A, PDPN). Each cell was scored for stemness activity using the function AddModuleScore in Seurat with default settings. A re-clustered heatmap of CNV scores of all epithelial cells was plotted using pheatmap (v1.0.12).

### 4.7 Pseudo-time analysis

The expression matrix and meta information of epithelial cells migrated from Seurat object were subsequently imported into R and analyzed using monocle3 (v1.0.0) (63) using the default parameters and a standard pipeline. After graph-autocorrelation analysis, the top 3,000 genes that varied with the trajectory were selected and collected into the gene module divided by pseudo-time. The graph of the change of key gene expression over the pseudo-time was drawn using the plot_genes_in_pseudo-time function.

### 4.8 Transcriptional regulator analysis

The specific transcriptional drivers in each epithelial cell subtype were analyzed by using the R package, SCENIC (v1.2.4) (64), under the guidance of a tutorial from https://github.com/aertslab/SCENIC. The binary score matrix of intracellular regulator activities was processed using *limma* (v3.48.3) (65) to find TFs that were differentially expressed in different cell subpopulations (logFC > 0 & p.adjust < 0.05), and the results were displayed using pheatmap (v1.0.12). The interaction network construction of high confidence dominating transcription factor and their target genes in epithelial cells and final visualization were implemented using Cytoscape (v3.9.1) (66).

In order to predict the possible functions of genes co-regulated by MYB and EN1, we used an online tool called Genemania (http://genemania.org/), which can find other genes that are related to a set of input genes by using a large set of genome and proteome association data. The result of GO enrichment analysis of above genes can be download from the website directly.

### 4.9 Cell–cell communication analysis

Intercellular communication within the tumor microenvironment was predicted using the python package, CellPhoneDB (v3.0.0) (67), based on the expression of interacting ligand and receptor genes. The number of iterations for the statistical analysis was set as 1,000. Significant (p < 0.05) mean results were further processed as follows. Cell pairs containing Duct/InterDuct/MEC were selected and divided into “source” and “target” groups according to the sequence of which in cell pairs. The expression scores of receptor–ligand pairs were considered as “counts” and constructed as a Seurat object and the differentially expressed receptor–ligand pairs in each group were revealed using the FindAllMarkers function with default parameters. Receptor–ligand pairs were selected for plot as those with p.adjust value <0.05, average log2FC >1. Heatmaps and dot plots were plotted using CellPhoneDB.

### 4.10 Expression program heterogeneity analysis

All epithelial cells were extracted from six samples and divided into three subgroups and the expression matrix was then normalized and decomposed using python package cNMF (v1.3) (68). The high-quality expression programs in each sample were manually selected, the Pearson’s correlation coefficients between them were calculated, and those with coefficients >0.18 and clustered together were considered as characteristic programs. In addition, correlation coefficients >0.6 were corrected to 0.6. The parameters, methods, and code used for data processing were adapted from a GitHub tutorial (https://github.com/dylkot/cNMF).

### 4.11 GSVA

GSVA was executed on hallmark gene sets, which were obtained from MSigDB (The Molecular Signatures Database) (69) and contained 50 well-defined pathways, using the R package, GSVA (v1.40.1) (70), with default settings. Differences between the pathway enrichment scores of the different groups was calculated by R package Limma (v3.48.3).

### 4.12 GO and KEGG enrichment analysis

GO and KEGG term enrichment analysis were performed using R packages clusterProfiler (v4.0.5) and org.Hs.eg.db (v3.13.0) with default settings (71).

### 4.13 Statistical analyses

The software, methods, and thresholds used for the statistical analyses are detailed in the Materials and Methods section. Wilcoxon test was used to reveal the statistical differences in **Figures 1E**, **2C** and **4B**. Two-tailed *t* test was used to reveal the statistical difference in **Supplementary Figures 5E, F**. A p.adjust-value or q-value <0.05 was considered significant.

## Data availability statement

The data presented in the study are deposited in the CNCB-NGDC (National Genomics Data Center, China National Center for Bioinformation, https://ngdc.cncb.ac.cn/gsa-human/s/j2QOEb3S), accession number is PRJCA012307.

## Ethics statement

The studies involving human participants were reviewed and approved by Ethics Committee Of Chinese PLA General Hospital. The patients/participants provided their written informed consent to participate in this study.

## Author contributions

Conceptualization: QX, HQ, XF. Surgery: QL, QX, FC, YR, LF, YS, FW, WY, HZ. Robotic assistant: HZ. Methodology: QX, HQ, XF, QL, ZJF. Pathology: ZS, WC. Data analysis: ZJF, HQ, CW, YS, YL, XF. Supervision: QX, HQ and XF. Writing—original draft: QL, ZJF. Writing—review & editing: QL, ZJF, JS, FC, YR. All authors contributed to the article and approved the submitted version.

## Funding

National Key Research and Development Program 2017YFB1304300 (HZ) National Key Research and Development Program 2020YFC2003405. National Natural Science Foundation of China Grant 81800939 (JS). Youth Incubation Program of Medical Science and Technology of PLA Grant 21QNPY114 (JS). The funders had no role in study design, data collection and analysis, decision to publish, or preparation of the manuscript.

## Acknowledgments

We thank all the patients for their generous donation of tissue samples for analysis in this study.

## Conflict of interest

The authors declare that the research was conducted in the absence of any commercial or financial relationships that could be construed as a potential conflict of interest.

## Publisher’s note

All claims expressed in this article are solely those of the authors and do not necessarily represent those of their affiliated organizations, or those of the publisher, the editors and the reviewers. Any product that may be evaluated in this article, or claim that may be made by its manufacturer, is not guaranteed or endorsed by the publisher.
