# Supplementary material for: Single-cell transcriptomic analysis of the tumor ecosystem of adenoid cystic carcinoma
Source: Front Oncol. 2022 Nov 17;12:1063477. doi: 10.3389/fonc.2022.1063477 (PMC9714264; doi:10.3389/fonc.2022.1063477)
Supplement: Supplementary file 1 [file DataSheet_1.pdf]

Single-cell transcriptomic analysis of the tumor ecosystem of adenoid cystic carcinoma

Quanquan Lin[1,4,†], Zhanjie Fang[2,3†], Jinlong Sun[4,†], Fei Chen[1,†], Yipeng Ren[1], Zhenhong Fu[1], Sefei Yang[1], Lin Feng[1], Feng Wang[4], Zhigang Song[1], Wei Chen[1], Wenjun Yu[1], Chen Wang[2,3], Yixin Shi[2,3], Yue Liang[2,3], Haizhong Zhang[1], Hongzhu Qu[2,3], Xiangdong Fang[2,3,*], Qing Xi[1*]*

[1.] Department of Stomatology, First Medical Center of PLA General Hospital, Beijing 100853, China.

[2.] CAS Key Laboratory of Genome Sciences and Information, Beijing Key Laboratory of Genome and Precision Medicine Technologies, Beijing Institute of Genomics, Chinese Academy of Sciences/China National Center for Bioinformation, Beijing, China

[3.] University of Chinese Academy of Sciences, Beijing, China

[4.] Department of Stomatology, Sixth Medical Center of PLA General Hospital, Beijing 100048, China.

† These authors contributed equally to this study.

* Corresponding authors:

Prof. Hongzhu Qu, E-mail: quhongzhu@big.ac.cn

Prof. Xiangdong Fang, E-mail: fangxd@big.ac.cn

Prof. Qing Xi, E-mail: xiqing301@163.com

Supplementary files

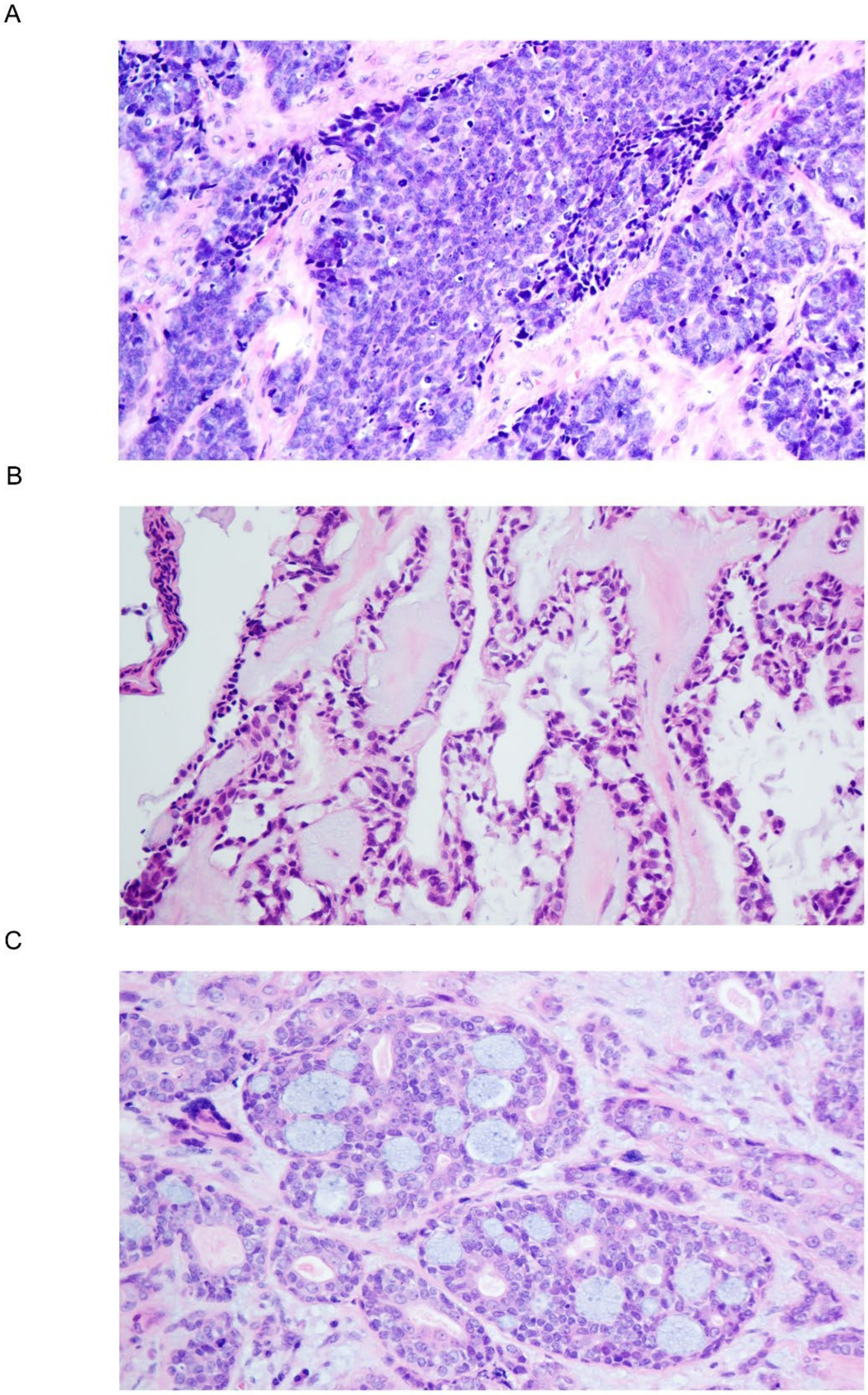

Figure S1. Staining pathologic sections of the tumor tissues of three patients were observed under a 400X microscope. (A) patient 0222 (B) patient 0329 (C) patient 7420

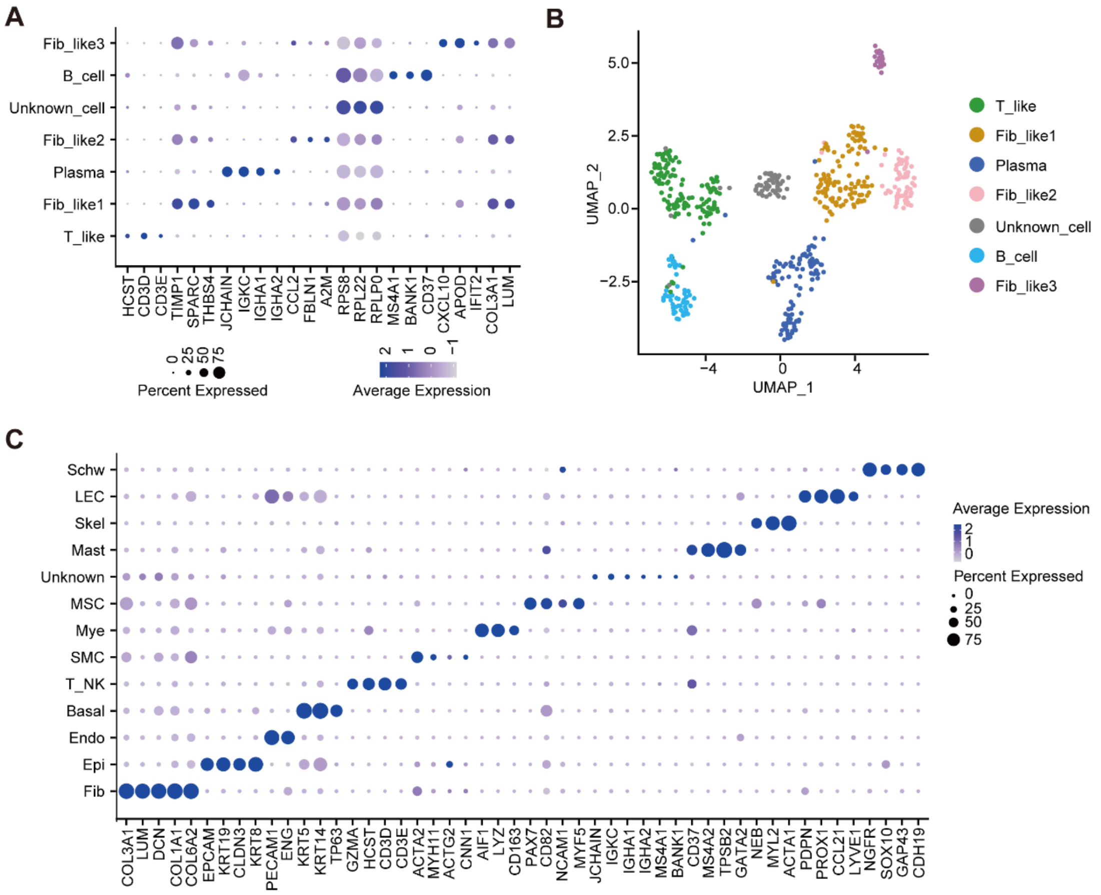

Figure S2. Relative expression of marker genes of different cell clusters. (A) Dot plot showing the relative expression of marker genes for various subtypes of undefined cell cluster. (B) UMAP plot shows seven cell populations reaggregated from “Unknown” cluster. (C) Dot plot showing the relative expression of marker genes for each main cell type.

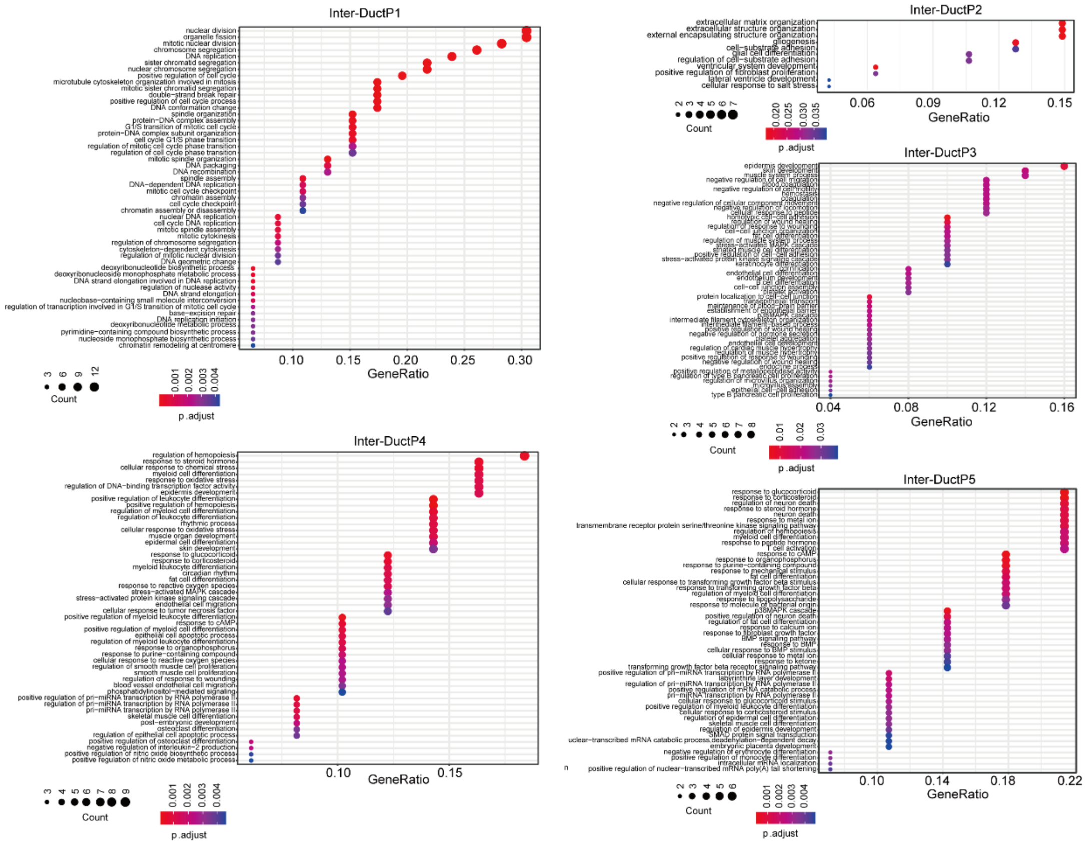

Figure S3. Dot plot showing the results of GO enrichment analysis for core gene sets of major expression programs in Inter-Duct cells.

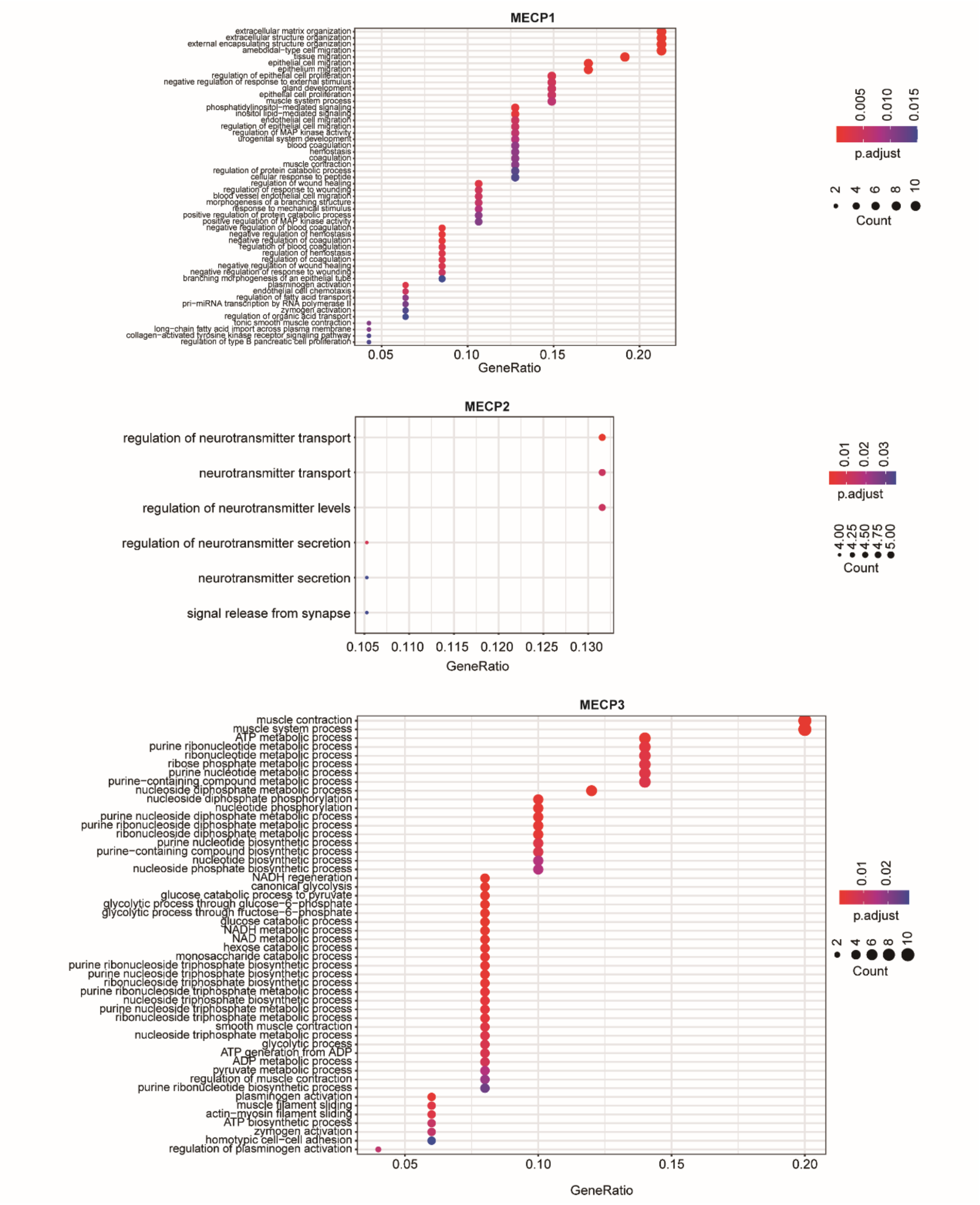

Figure S4. Dot plot showing the results of GO enrichment analysis for core gene sets of major expression programs in myoepithelial-like cells.

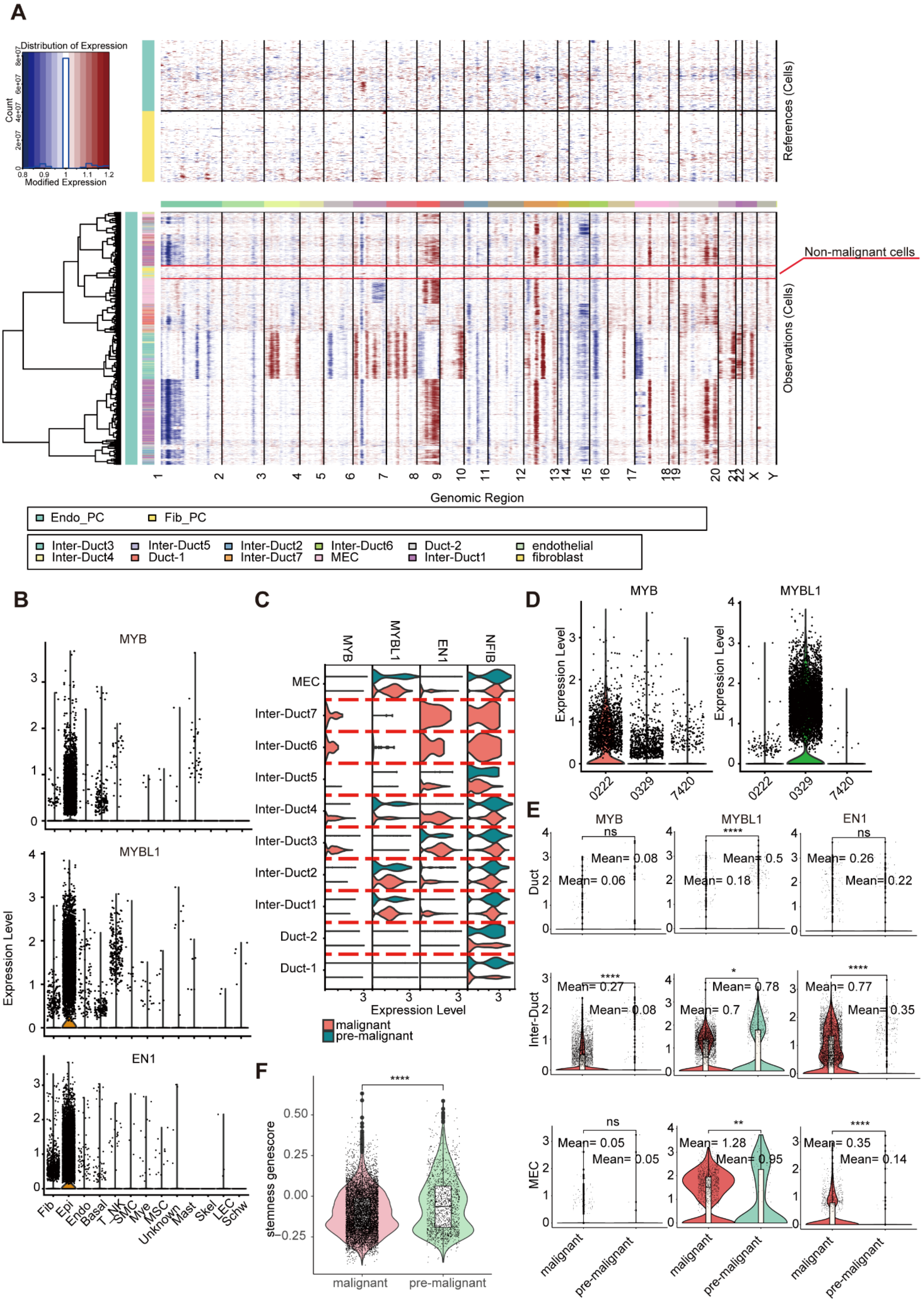

A
Distribution of Expression
Count
Modified Expression
References (Cells)
Observations (Cells)
Non-malignant cells
Genomic Region
Endo_PC
Fib_PC
Inter-Duct3
Inter-Duct4
Inter-Duct5
Duct-1
Inter-Duct2
Inter-Duct7
Inter-Duct6
MEC
Duct-2
Inter-Duct1
endothelial
fibroblast
B
MYB
MYBL1
EN1
Expression Level
Fib
Epi
Endo
Basal
T_NK
SMC
Mye
MSC
Unknown
Mast
Skel
LEC
Schw
C
MYB
MYBL1
EN1
NFIB
MEC
Inter-Duct7
Inter-Duct6
Inter-Duct5
Inter-Duct4
Inter-Duct3
Inter-Duct2
Inter-Duct1
Duct-2
Duct-1
Expression Level
malignant
pre-malignant
D
MYB
MYBL1
Expression Level
0222
0329
7420
E
MYB
MYBL1
EN1
Duct
Inter-Duct
MEC
Mean= 0.08
Mean= 0.06
Mean= 0.5
Mean= 0.18
Mean= 0.26
Mean= 0.22
Mean= 0.27
Mean= 0.08
Mean= 0.78
Mean= 0.7
Mean= 0.77
Mean= 0.35
Mean= 0.05
Mean= 0.05
Mean= 1.28
Mean= 0.95
Mean= 0.35
Mean= 0.14
ns
****
*
**
malignant
pre-malignant
F
stemness genescore
malignant
pre-malignant

Figure S5. Identification of malignant epithelial cells.

(A) Inferred large-scale chromosomal copy number alterations were used to discern malignant cells. Nonmalignant cells were identified and labeled on the right side of the figure. (B)Violin plots showing the expression of MYB (upper), MYBL1 (middle), and EN1 (lower) in different cell types. (C) Violin plot showing the expression of MYB/MYBL1/EN1/NFIB in different epithelial cell subtypes. Malignant states are distinguished by different colors. (D) Violin plot showing the expression of MYB/MYBL1 in epithelial cells from different patients. (E) Stemness geneset scores were calculated for "malignant" and "premalignant" groups. All p-values were calculated by two-tailed *t* test. **** p $\leq$ 0.0001. (F) Violin plot showing the expression of MYB/MYBL1/EN1 in three main epithelial cell subtypes between "malignant" and "premalignant" groups. All p-values were calculated using two-tailed t test. ns, $p > 0.05$, * $p \leq 0.05$, ** $p \leq 0.01$, and **** $p \leq 0.0001$

Supplementary Table 1. Quality control information of each sample.

Patient ID	Sample Source	Estimated Number of Cells	Median Genes per Cell	Sequencing Saturation (%)	Number of cells after QC
0222	Para-carcinoma	9,962	3022	42.3%	8454
0222	Carcinoma	6,958	3,334	56.2%	6306
0329	para-carcinoma	8,277	2,780	64.3%	7512
0329	Carcinoma	6,633	2,674	51.8%	5205
7420	para-carcinoma	10,012	1,509	72.9%	8227
7420	Carcinoma	8,106	1,718	65.2%	7010

Supplementary Table 2. Detailed clinical information of each patient.

Sample	Age	Sex	Foci	Pathology
0222	65	Male	Right buccal mucosal	Solid type
0329	51	Female	Left mouth floor	Cribriform type
7420	55	Female	Left tongue	Cribriform type

Supplementary Table 3. Dominant genes in each expression program of three main epithelial subtypes.

Duct		
**P1**	**P2**	**P3**
SMARCD3	MUL1	PTGFR
BX322562.1	CHD1L	ADM
PRAME	ZC3H11A	LMO3
SHLD1	CSTA	JAZF1
SUPT7L	ADAMTS2	ARHGAP31
PWWP2B	BBS9	USP45
HIGD1B	AL158206.1	DCLRE1C
MUC16	AC132192.2	ZC3H10
IL1R2	LINC02762	PHETA1
ASH1L.AS1	SLC37A4	AK5
AC099332.1	JHY	DENND4B
MIR3142HG	GPR180	DNM3OS
TRAM2.AS1	DMXL2	COLGALT2
AC025031.4	CYGB	DUSP19
CPB2.AS1	LYL1	EPHA3
C17orf67	TRIB3	SLC49A3
SSTR2	AL034417.3	ENPEP
KRTDAP	GJA4	HAND2.AS1
EML2	CSF1	KCNK17
GSPT2	NFASC	FAM135A
ATAD3C	AC092164.1	LINC00997
LINC01137	HAAO	ADAM22
LURAP1	TNS1	GPC2
WDR63	GHRLOS	ASTN2
ASTN1	SLC6A1	PNPLA7
GOLT1A	AC092903.2	FAM180B
LYPLAL1.DT	CTBP1.DT	SLC16A7
LINC01703	ADAMTS3	ZC2HC1C
PLGLB2	ABCG2	SETD1A
GCC2.AS1	CDH6	AC092140.2
RGPD8	NRG2	BORCS6
AC006460.2	ME1	CDK5R1
MAATS1	RPS6KA2	PIEZO2
CAMK2N2	PDE1C	PCSK4
AC072022.2	SRRM3	EVI5L
LINC00989	ZSCAN25	ARHGAP33
LRIT3	TTLL11	VSIG10L
FAM218A	LCN1	ZNF446
AC107214.1	VSTM4	PPP1R3D

ANKRD33B	IFIT2	VPREB3		
AC099522.2	ENTPD1	SLC35E4		
ARRDC3.AS1	INA	AL357568.1		
AC010226.1	MICAL2	KBTBD8		
ZFP62	TPCN2	TMEM154		
ZNF391	ABCC9	FAM124A		
AL662884.3	BCAT1	FAM81A		
BMP5	ACVRL1	NOMO3		
OOEP	PXN.AS1	AC093462.1		
KIAA0408	STARD13	LENG8.AS1		
AL121933.2	SMAD9	TOX2		

Inter_Duct				
**P1**	**P2**	**P3**	**P4**	**P5**
TUBA1B	VCAN	GPRC5A	ZFP36	HBA1
RRM2	EFHD1	PLAUR	FOSB	AC006387.1
PCLAF	NFIB	EGR3	JUNB	CIRBP.AS1
CDK1	FRMD4A	LMNA	PPP1R15A	TMEM81
H2AFZ	VGLL4	ZNF750	BTG2	AC244453.2
FEN1	MGP	YWHAZ	ATF3	GDF15
MCM10	HNRNPA1	TM4SF1	FOS	ACTL10
TUBB	SERPINE2	KRT17	EGR3	CD274
TYMS	BCL2	EZR	ZFP36L1	AC027020.2
NPB	ITGA6	CLDN4	NFKBIA	AC010359.2
MYBL2	FABP7	KLF6	MAFF	CYB561D2.1
C1orf54	SRPK2	KDM6B	NR4A1	SPACA6P.AS
ARHGAP24	PBX1	DSP	TNFAIP3	LINC00310
DUT	RASA1	KLF5	LMNA	SNX20
TK1	MFGE8	KRT8	KLF6	LINC02328
MAD2L1	CDK6	ITPKC	BHLHE40	ZNF540
ATAD2	NRCAM	SHC4	FTH1	CNTNAP3C
KNTC1	LRIG1	CLIP1	NR4A2	WNT1
DIAPH3	MYB	EMP1	NFKBIZ	ZNF578
AC027682.6	ADGRG6	NR4A1	JUN	AL359878.1
HMGN2	LDLRAD4	SAT1	CLDN4	AL049779.4
RACGAP1	NREP	NR4A3	EFNA1	AC107029.2
CENPK	AZGP1	VMP1	CLDN3	AC131212.1
HMGB1	ART3	SERPINB5	KRT17	AL109955.1
TPX2	SMARCC1	IRF2BP2	SAT1	AC074044.1
PCNA	PRLR	EEF1A1	NCOA7	AC120024.1
PSMC3IP	CD44	RAB3IP	EZR	AL096701.4
HIST1H4C	MFAP3L	PMAIP1	PTX3	FOS
MCM7	CSRP2	INHBA	CRYAB	TNFRSF18
HMGB2	SEMA6D	EDN1	TIPARP	JUNB
MKI67	AQP1	WWTR1	TMSB10	JUN

STAT4	COL27A1	RAP2B	MSLN	ZFP36
NUSAP1	NAP1L4	FOSL1	IER3	IER2
HELLS	FAT1	KRT16	NAMPT	EGR1
CKS1B	VIT	PDE4B	ANXA3	IER3
RGS5	HNRNPA3	MAP3K8	ELF3	BTG2
CLSPN	FLRT2	SPAG9	KLF10	DDIT4
STMN1	SLC35F3	TGIF1	TPT1.AS1	FOSB
DEK	SCRG1	VCL	TPM1	AC132938.6
RANBP1	ZKSCAN1	TPM4	ETS2	DUSP1
HJURP	VIM	GADD45A	CCNL1	HES1
CDCA8	LDHB	ZFAND5	KLF4	GADD45B
FN1	SPARC	JUND	RASGEF1B	RASD1
ESCO2	TM4SF18	RAB11FIP1	MIA	SLC30A4
TMPO	SPPL3	KLF3	PMEPA1	NFKBIA
TACC3	ABCA13	CLDN3	KRT8	ADPRH
MCM3	AC092683.1	ACTG1	CSRNP1	AP000442.1
FOXM1	MICAL3	ZFP36L1	MAP3K8	ZFP36L1
BTN3A3	CRABP1	IL27RA	KRT6B	PPP1R15A
KIF23	CAPS	NCOA7	TNF	NR4A1

**MEC**		
**P1**	**P2**	**P3**
ARID5B	CYP2S1	TPM2
KLF6	AC000065.2	MYL9
THBS1	PRAG1	ACTA2
RCAN1	LPL	MYL6
GADD45A	AC068189.2	TAGLN
CALD1	APBA1	TPM1
LDLR	CCDC180	SAA1
TACSTD2	BARX2	NME2
TPM4	CLEC14A	C5orf46
SERPINB5	ITGB3	MYH11
TRIB1	TNFRSF14.AS1	MGST3
ERRFI1	NCDN	LGALS1
TES	ARTN	PGK1
RTN4	LPAR3	ENO1
EDN1	AKNAD1	DALRD3
TM4SF1	NHLH2	CTSZ
NEDD4L	ECM1	MSRB3
RAP2B	SYT11	NDUFA13
EZR	ZBED6	TGFB1I1
COL4A1	ABCB10	MGST1
CSNK1A1	DGUOK.AS1	GAPDH
COL4A2	STK39	ARPC3
EGR3	LINC00852	SERF2

GLIPR1	NUP210	JOSD2
SERPINE2	ATP13A5	PRDX5
A2M	AC110792.3	FOLR1
ADAMTS1	EPB41L4A	UQCR11
KRT17	STK32A	MYL12B
AL161431.1	CAMK2A	ETFB
MYLK	ETV7	TSTD1
ETS1	RIMS1	ANXA3
AC016831.1	HMGN3.AS1	BLVRB
AMOTL2	AL365259.1	PIGT
COL9A3	AL356417.2	MRPL36
IRS2	PDE1C	CD151
COL9A2	DPY19L1	TNFRSF14
DDX5	ZPBP	LAPTM5
DUSP6	CFAP69	SNRNP40
MIR222HG	RASA4	GUK1
CNN3	MGAM	FIBP
COL9A1	AC044893.1	KRT7
PLCB4	OPLAH	AAMDC
NCOA7	IGFBPL1	MYLK
ACTG2	SVEP1	ABCC5
MYC	VAV2	EBP
NR4A1	IL15RA	ATP5MC3
NTRK3	AGAP9	PKM
SRSF5	STK33	PPP1R14A
KLF5	SYTL2	PLEKHJ1
PLAT	ME3	CNN1

Supplementary Table 4. **Genes belonging to module 5 and 13**.

Module 5 geneset				
id	module	supermodule	dim_1	dim_2
GPR153	5	1	-5.007205	1.5422076
RERE	5	1	-2.82665	2.2635716
SPSB1	5	1	-3.21565	1.9036111
LZIC	5	1	-3.756542	2.0042471
TARDBP	5	1	-3.115615	2.0939163
DDI2	5	1	-3.178537	2.0202283
RCC2	5	1	-3.900816	1.7036618
KDM1A	5	1	-4.272929	1.4908394
RCAN3	5	1	-3.667017	1.6590899
WASF2	5	1	-3.596046	1.9667777
ZBTB8B	5	1	-4.956421	1.4666773
RBBP4	5	1	-3.604729	1.9415025
PHC2	5	1	-4.553259	1.0468336
AGO1	5	1	-3.138649	2.0039724
PABPC4	5	1	-3.665015	1.8723391
GPBP1L1	5	1	-3.369774	1.9799169
TTC39A	5	1	-3.97107	1.568813
PRPF38A	5	1	-3.543763	1.8783063
KANK4	5	1	-4.146931	2.3718246
S100A9	5	1	-5.181973	1.5193845
S100A8	5	1	-5.231831	1.5189301
MPZL1	5	1	-3.455937	2.2805208
ERVMER61-1	5	1	-4.737498	1.3349646
SERTAD4	5	1	-2.712389	2.2864012
TGFB2	5	1	-4.854412	1.5047871
SLC35F3	5	1	-3.999684	2.266034
EXOC6B	5	1	-3.978457	1.9512261
FAM178B	5	1	-4.320481	1.5325292
MGAT4A	5	1	-4.827541	1.3504173
LINC02611	5	1	-5.015086	1.5026635
EN1	5	1	-4.454297	0.967575
FMNL2	5	1	-3.992871	1.6253981
ITGA6	5	1	-4.166293	2.0894817
RAPGEF4	5	1	-4.681777	1.3053757
TTLL4	5	1	-4.420017	1.4512307
EFHD1	5	1	-4.604629	1.22722
ATG7	5	1	-4.744643	1.172649
NR1D2	5	1	-2.876824	2.1554068
TOP2B	5	1	-3.407952	1.8408083
ACVR2B	5	1	-4.390508	1.4872242
ZNF621	5	1	-2.465129	2.2064542

AC099541.2	5	1	-4.978692	1.4868962
CTNNB1	5	1	-3.285813	2.231306
NME6	5	1	-4.019914	1.05378
CHDH	5	1	-4.252699	1.6139318
CCDC66	5	1	-3.41081	1.6009959
ARHGEF3	5	1	-4.445832	1.427595
IL17RD	5	1	-4.58462	1.4488334
PDE12	5	1	-4.397863	1.2384266
LRIG1	5	1	-4.563129	1.5441119
GSK3B	5	1	-2.934169	2.1188668
H1FX	5	1	-4.606097	0.9563474
RYK	5	1	-3.169559	1.8954567
TFDP2	5	1	-3.137192	1.7594217
GK5	5	1	-4.20401	1.3906833
TM4SF18	5	1	-4.756154	1.1935412
PLCH1	5	1	-3.486033	1.6157309
TBL1XR1	5	1	-3.33702	2.0481557
PIK3CA	5	1	-2.759402	2.2055239
DLG1	5	1	-3.206873	1.9921879
SPON2	5	1	-4.5845	0.9754969
MAEA	5	1	-3.983234	2.2789735
APBB2	5	1	-4.266006	1.5019847
KIT	5	1	-4.020064	1.2749242
REST	5	1	-2.760432	2.0644535
RUFY3	5	1	-2.556882	2.2678713
CCNG2	5	1	-2.595337	2.351993
BMPR1B	5	1	-4.649308	1.4646355
PDGFC	5	1	-4.045438	2.3421191
MFAP3L	5	1	-4.284775	1.6136965
SAP30	5	1	-4.630625	0.9469487
PDZD2	5	1	-5.018226	1.5481831
PRLR	5	1	-3.448149	1.6004168
RASA1	5	1	-4.066605	2.3117761
LINC00461	5	1	-4.662994	1.1975507
STARD4-AS1	5	1	-4.146231	2.3387889
NREP	5	1	-4.110378	2.3546819
DCP2	5	1	-3.191216	1.9190339
PITX1	5	1	-4.646805	0.9024011
KDM3B	5	1	-3.665735	1.8239612
CNOT6	5	1	-3.097434	1.8392352
PRPF4B	5	1	-3.072692	2.0606841
CDYL	5	1	-4.168427	1.1472954
ID4	5	1	-4.61147	1.1837684
C6orf62	5	1	-2.849271	2.1920384

HIST1H2BH	5	1	-4.56027	0.9446687
ATF6B	5	1	-4.35436	1.4806391
CLPSL1	5	1	-4.913615	1.303183
STK38	5	1	-3.543613	1.5638484
C6orf89	5	1	-2.896017	2.1379861
FAM83B	5	1	-2.648303	2.3880843
PHIP	5	1	-3.394288	1.8266577
PM20D2	5	1	-4.483912	1.0150226
AK9	5	1	-4.292574	1.4229971
HINT3	5	1	-4.395828	0.9697648
PTPRK	5	1	-3.976921	1.3432177
EPB41L2	5	1	-4.485825	1.8004884
CCN2	5	1	-4.116644	2.3757304
**MYB**	5	1	-4.7192	1.3989991
ADGRG6	5	1	-4.796855	1.4459082
SASH1	5	1	-4.322892	1.3569332
TAB2	5	1	-2.929214	2.1807588
FNDC1	5	1	-4.150895	2.3879603
QKI	5	1	-2.830342	2.3011249
AFDN	5	1	-3.979236	1.2973124
PHF10	5	1	-3.862192	1.7141758
CYTH3	5	1	-4.332113	1.5111817
AHR	5	1	-4.201499	1.1456226
CDCA7L	5	1	-4.448309	0.8447551
SNHG26	5	1	-4.363199	0.8925945
MTURN	5	1	-3.24055	1.8991994
AQP1	5	1	-4.096154	1.8568362
ZNF727	5	1	-4.279354	1.2735931
ZNF736	5	1	-3.506125	1.7317416
ZNF138	5	1	-4.057035	1.2413768
ZNF117	5	1	-3.137873	2.0043768
GTF2I	5	1	-2.86094	2.1402797
FZD1	5	1	-3.208602	2.0313739
GATAD1	5	1	-3.066974	1.9799966
ZNF3	5	1	-4.034411	1.2364358
PILRB	5	1	-3.128263	1.8619508
BCAP29	5	1	-3.481762	2.0226425
LAMB1	5	1	-4.143023	2.393185
MEST	5	1	-4.669441	1.1628171
AUXG01000058.1	5	1	-4.431668	1.6975595
UBN2	5	1	-2.930023	2.1769503
DENND11	5	1	-4.236858	1.2228202
RBM33	5	1	-2.469388	2.3107528
WDR60	5	1	-2.703686	2.2859363

PIWIL2	5	1	-4.569514	1.0293099
PPP3CC	5	1	-4.703323	1.1827651
NCALD	5	1	-4.96128	1.4727126
SMARCA2	5	1	-2.550201	2.3242515
KIAA2026	5	1	-2.823282	2.1673039
UHRF2	5	1	-4.213363	1.4152583
ELAVL2	5	1	-4.734396	1.4840568
DCAF10	5	1	-2.800458	2.148868
VPS13A	5	1	-4.201097	1.8364033
GNAQ	5	1	-2.982661	2.1381425
LINC01505	5	1	-4.140588	2.4007906
PALM2-AKAP2	5	1	-4.443748	1.9079975
ZNF618	5	1	-4.123237	2.3695558
RC3H2	5	1	-3.306551	1.7705501
NCS1	5	1	-4.103138	2.2715863
PRRC2B	5	1	-2.941751	2.2082404
TSC1	5	1	-2.949838	2.0942873
REXO4	5	1	-4.161279	1.0491549
BRD3	5	1	-3.426421	1.5497002
ENTR1	5	1	-4.389381	1.0838865
ZMYND19	5	1	-4.594834	1.0555295
UPF2	5	1	-2.8108	2.1517862
BEND7	5	1	-4.132354	1.2723242
FRMD4A	5	1	-3.966058	1.3593315
PLXDC2	5	1	-3.577702	2.0491222
ARHGAP21	5	1	-2.912816	2.2669343
PARD3	5	1	-3.631675	2.0758299
TM9SF3	5	1	-2.906711	2.1368112
NAV2	5	1	-3.985026	1.6917664
CCDC34	5	1	-4.491557	0.8060752
LGR4	5	1	-3.775674	2.1219834
DTX4	5	1	-4.118052	1.6355654
SNHG1	5	1	-4.292886	1.1187307
LINC02747	5	1	-4.787698	1.3374983
FAM181B	5	1	-4.979877	1.5113853
ST3GAL4	5	1	-4.004677	2.291686
ATN1	5	1	-2.99245	1.8407558
ATF7IP	5	1	-2.622935	2.1854142
ST8SIA1	5	1	-4.952324	1.4929115
DIP2B	5	1	-3.168807	1.9039921
KRT6C	5	1	-5.224986	1.5099327
KRT6A	5	1	-5.227731	1.4955114
MDM1	5	1	-4.474395	1.2001549
NUP107	5	1	-4.409424	1.0389301

SLC35E3	5	1	-4.502388	1.3247763
MDM2	5	1	-4.220016	1.3537005
CPM	5	1	-4.818882	1.3825678
CPSF6	5	1	-4.336782	1.4564454
YEATS4	5	1	-4.425244	0.7840473
FRS2	5	1	-4.309532	1.2044972
CNOT2	5	1	-4.165814	1.2437931
ZFC3H1	5	1	-3.416994	1.8494228
RAB21	5	1	-4.100205	1.1465179
TBC1D15	5	1	-4.058944	1.1687082
ATXN7L3B	5	1	-4.060133	1.1813294
PAWR	5	1	-4.082912	1.3839125
PPP1R12A	5	1	-3.615417	2.1495613
LRRIQ1	5	1	-4.604085	1.3296851
SLC5A8	5	1	-4.965389	1.4970039
RFLNA	5	1	-4.710429	1.2834653
FBRSL1	5	1	-4.297555	1.1189207
RBM26	5	1	-2.67187	2.291057
RAD51B	5	1	-4.559341	1.1014794
CIPC	5	1	-4.233326	1.407267
FOXN3	5	1	-2.626844	2.3421921
MTA1	5	1	-3.927812	1.2175194
FAM98B	5	1	-3.617331	1.5946568
BMF	5	1	-4.682224	1.4922387
OIP5-AS1	5	1	-4.148583	1.0595015

Module 13 geneset

id	module	supermodule	dim_1	dim_2
KLHL21	13	1	3.720566	-0.291338
IFFO2	13	1	2.9059997	-0.284772
STX12	13	1	3.8137643	0.1324395
ZC3H12A	13	1	3.3741875	-0.429461
TACSTD2	13	1	3.9132469	-0.225617
GADD45A	13	1	3.8486478	-0.086036
GTF2B	13	1	3.7892549	-0.094294
RHOC	13	1	5.0010023	0.3855158
S100A10	13	1	4.5897138	0.1623436
S100A11	13	1	4.5433073	0.1141657
IL6R	13	1	3.8834577	0.1021332
LMNA	13	1	4.097357	0.2780219
IFI16	13	1	3.8922217	-0.426862
LAMC2	13	1	3.8699086	0.0684818
PRELP	13	1	4.529804	-0.273521
B3GALNT2	13	1	3.8822811	0.1255213
PXDN	13	1	3.8478033	-0.171736

ZFP36L2	13	1	3.2684581	0.0230812
RTN4	13	1	3.7207031	-0.136092
CCDC88A	13	1	4.2833073	-0.456485
REL	13	1	3.2625966	-0.26199
PAX8-AS1	13	1	4.4043956	-0.400622
NFE2L2	13	1	3.7712405	-0.504565
COQ10B	13	1	3.5714109	-0.148097
NRP2	13	1	4.4067628	-0.334202
IGFBP2	13	1	4.4850628	-0.360169
CSRNP1	13	1	3.9406712	0.029765
GNAI2	13	1	4.399502	0.0525321
FRMD4B	13	1	4.2538686	-0.51445
ATP1B3	13	1	3.7747917	-0.367092
RAP2B	13	1	3.7098465	-0.103674
TIPARP	13	1	3.3140616	-0.036759
RNF212	13	1	4.7741084	0.2266089
CXCL2	13	1	3.2391179	-0.45979
NFKB1	13	1	3.5657096	-0.190008
SGMS2	13	1	3.7167752	-0.017353
SLC25A4	13	1	4.6830943	0.1503612
BASP1	13	1	4.2822854	-0.490105
IL6ST	13	1	3.8713958	-0.538249
ZSWIM6	13	1	3.6418149	-0.220384
HAPLN1	13	1	4.4681287	-0.363765
ELL2	13	1	3.6105592	-0.1592
PDLIM4	13	1	4.0606534	-0.328315
IRF1	13	1	3.2473912	-0.529502
SQSTM1	13	1	3.2424314	-0.417051
TUBB2A	13	1	3.9647028	0.064643
IER3	13	1	3.2151942	-0.379748
HSPA1A	13	1	3.0018452	-0.249642
CDKN1A	13	1	3.9549029	0.0255028
MDFI	13	1	4.9713964	0.4345573
TNFAIP3	13	1	3.2565675	-0.378565
CREB5	13	1	3.8109405	-0.098689
IFRD1	13	1	3.2375796	0.0459958
AC016831.1	13	1	3.8117087	-0.178223
LINC-PINT	13	1	4.0186164	0.0516347
CLDN23	13	1	4.7943461	0.2790848
TUSC3	13	1	4.4951351	0.1317462
DPYSL2	13	1	4.7681055	0.2223274
SARAF	13	1	4.3348429	0.0339893
FGFR1	13	1	4.3688035	-0.45051
TACC1	13	1	4.2578108	-0.396156

SFRP1	13	1	4.3570828	-0.470732
CEBPD	13	1	4.0082362	0.1033267
SDCBP	13	1	4.4363732	-0.018793
**MYBL1**	13	1	4.4569249	-0.36704
MTDH	13	1	4.2206163	0.1081911
ZNF706	13	1	4.6660819	0.527574
KLF10	13	1	3.9601521	0.0192369
ENY2	13	1	4.6580024	0.2152389
TRIB1	13	1	3.4004333	0.0645021
EFR3A	13	1	4.3025515	-0.489963
EEF1D	13	1	4.9525278	0.2824897
PLEC	13	1	4.1878962	-0.350688
GPAA1	13	1	4.709902	0.0696095
CYC1	13	1	4.6903825	0.1216418
RPL8	13	1	5.0059008	0.3560914
CCDC107	13	1	4.5044489	0.165172
CEMIP2	13	1	3.9281764	0.0083779
CARD19	13	1	4.6900494	0.3974995
STOM	13	1	3.6979873	-0.408961
PFKFB3	13	1	3.1547251	-1.020911
VIM	13	1	4.7716844	0.1003162
ARID5B	13	1	3.8383539	-0.142462
P4HA1	13	1	4.3515441	-0.439533
VCL	13	1	3.6021776	-0.443788
ANXA11	13	1	3.4663477	-0.743101
BAG3	13	1	3.5271497	-0.527456
CAVIN3	13	1	4.4859979	0.0254136
RIC3	13	1	4.4284	0.0730292
LDHA	13	1	4.5470278	-0.042684
C1QTNF4	13	1	4.7975111	0.1457826
FTH1	13	1	4.970619	0.3089277
SLC3A2	13	1	3.6883321	-0.144568
NEAT1	13	1	2.9580029	-0.51404
CCDC85B	13	1	5.1422577	0.6294853
NECTIN1	13	1	3.6910269	-0.310217
TRIM29	13	1	3.9638178	-0.291421
CLMP	13	1	4.3141217	-0.420382
ETS1	13	1	3.7445648	-0.317785
CCND2	13	1	4.1733022	-0.4893
CD9	13	1	3.8367519	-0.376476
TUBA1C	13	1	3.7770376	-0.34942
KRT81	13	1	4.4602735	0.1340639
GLIPR1	13	1	3.789026	-0.143263
ATP2B1	13	1	3.945061	-0.474545

WDR66	13	1	4.7213695	0.2598559
UBC	13	1	3.8944349	-0.044425
IRS2	13	1	3.7177784	-0.057505
FAM177A1	13	1	3.6919773	-0.366743
NFKBIA	13	1	3.2044527	-0.289626
FERMT2	13	1	3.8373141	-0.346454
ARG2	13	1	4.033334	0.0366522
SRSF5	13	1	3.7417688	-0.315991
CALM1	13	1	4.1308024	-0.012409
SERPINA3	13	1	4.8815207	0.4880475
ANXA2	13	1	3.5457418	-0.967849
